# Survival of children with rare structural congenital anomalies: a multi-registry cohort study

**DOI:** 10.1186/s13023-022-02292-y

**Published:** 2022-03-29

**Authors:** Alessio Coi, Michele Santoro, Anna Pierini, Judith Rankin, Svetlana V. Glinianaia, Joachim Tan, Abigail-Kate Reid, Ester Garne, Maria Loane, Joanne Given, Elisa Ballardini, Clara Cavero-Carbonell, Hermien E. K. de Walle, Miriam Gatt, Laura García-Villodre, Mika Gissler, Sue Jordan, Sonja Kiuru-Kuhlefelt, Stine Kjaer Urhoj, Kari Klungsøyr, Nathalie Lelong, L. Renée Lutke, Amanda J. Neville, Makan Rahshenas, Ieuan Scanlon, Diana Wellesley, Joan K. Morris

**Affiliations:** 1 Unit of Epidemiology of Rare Diseases and Congenital Anomalies, Institute of Clinical Physiology, National Research Council, Via Moruzzi 1, 56124 Pisa, Italy; 2 Fondazione Toscana Gabriele Monasterio, Pisa, Italy; 3 Population Health Sciences Institute, Faculty of Medical Sciences, Newcastle University, Newcastle upon Tyne, UK; 4 Population Health Research Institute, St George’s University of London, London, UK; 5 Paediatric Department, Hospital Lillebaelt, Kolding, Denmark; 6 Faculty of Life and Health Sciences, Ulster University, Belfast, Northern Ireland, UK; 7 Neonatal Intensive Care Unit, Paediatric Section, IMER Registry (Emilia Romagna Registry of Birth Defects), Department of Medical Sciences, University of Ferrara, Ferrara, Italy; 8 Rare Diseases Research Unit, Foundation for the Promotion of Health and Biomedical Research in the Valencian Region, Valencia, Spain; 9 Department of Genetics, University Medical Center Groningen, University of Groningen, Groningen, The Netherlands; 10 Malta Congenital Anomalies Register, Directorate of Health Information and Research, Pieta, Malta; 11 Information Services Department, THL Finnish Institute for Health and Welfare, Helsinki, Finland; 12 Faculty of Medicine, Health and Life Science, Swansea University, Swansea, Wales, UK; 13 Section of Epidemiology, Department of Public Health, University of Copenhagen, Copenhagen, Denmark; 14 Department of Global Public Health and Primary Care, University of Bergen, Bergen, Norway; 15 Division for Mental and Physical Health, Norwegian Institute of Public Health, Bergen, Norway; 16 Center of Research in Epidemiology and StatisticS/CRESS/Obstetrical Perinatal and Pediatric Epidemiology Research Team (EPOPé), INSERM, INRA, Université de Paris, Paris, France; 17 IMER Registry (Emila Romagna Registry of Birth Defects), Center for Clinical and Epidemiological Research, University of Ferrara Azienda Ospedaliero- Universitaria di Ferrara, Ferrara, Italy; 18 Wessex Clinical Genetics Service, Princess Anne Hospital, Faculty of Medicine, University Hospital Southampton, Southampton, UK

## Abstract

**Background:**

Congenital anomalies are the leading cause of perinatal, neonatal and infant mortality in developed countries. Large long-term follow-up studies investigating survival beyond the first year of life in children with rare congenital anomalies are costly and sufficiently large standardized cohorts are difficult to obtain due to the rarity of some anomalies. This study aimed to investigate the survival up to 10 years of age of children born with a rare structural congenital anomaly in the period 1995–2014 in Western Europe.

**Methods:**

Live births from thirteen EUROCAT (European network for the epidemiological surveillance of congenital anomalies) population-based registries were linked to mortality records. Survival for 12,685 live births with one of the 31 investigated rare structural congenital anomalies (CAs) was estimated at 1 week, 4 weeks and 1, 5 and 10 years of age within each registry and combined across Europe using random effects meta-analyses. Differences between registries were evaluated for the eight rare CAs with at least 500 live births.

**Results:**

Amongst the investigated CAs, arhinencephaly/holoprosencephaly had the lowest survival at all ages (58.1%, 95% Confidence Interval (CI): 44.3–76.2% at 1 week; 47.4%, CI: 36.4–61.6% at 1 year; 35.6%, CI: 22.2–56.9% at 10 years). Overall, children with rare CAs of the digestive system had the highest survival (> 95% at 1 week, > 84% at 10 years). Most deaths occurred within the first four weeks of life, resulting in a 10-year survival conditional on surviving 4 weeks of over 95% for 17 out of 31 rare CAs. A moderate variability in survival between participating registries was observed for the eight selected rare CAs.

**Conclusions:**

Pooling standardised data across 13 European CA registries and the linkage to mortality data enabled reliable survival estimates to be obtained at five ages up to ten years. Such estimates are useful for clinical practice and parental counselling.

## Background

Congenital anomalies (CA) affect approximately 3% of births in Europe and in the United States [1, 2]. CAs, including structural defects, chromosomal anomalies, and genetic syndromes, are the leading cause of perinatal, neonatal and infant mortality in developed countries [3–5].

Advances in neonatal and paediatric care have led to an overall improvement in survival of children with CAs beyond infancy [6, 7]. However, large long-term follow-up studies investigating survival beyond the first year of life in children with rare CAs are costly and time-consuming; therefore, such research is scarce and little is known about the long-term outcomes of children born with certain rare CAs. Published results mainly refer to case series or hospital cohorts often estimating mortality at a point in time and very rarely starting from birth [8–14]. Due to the rarity of some CAs, sufficiently large standardized cohorts are difficult to obtain and the only way to accurately study survival in children with these CAs is to pool data across several registries and link cases to mortality databases [15, 16]. Pooling CA data from registries across Europe using standardized definitions and classification of CAs provides the opportunity to produce reliable survival estimates for children with rare CAs and a rich dataset for future research.

This study aimed to investigate the survival up to 10 years of age of children born with a rare structural CA in the period 1995–2014 using data from 13 EUROCAT (European network for the epidemiological surveillance of CAs) registries. The study is part of the EUROlinkCAT project that linked data of live born children with CAs to mortality data sources and other electronic administrative, healthcare and education databases to investigate the survival, morbidity and educational outcomes up to 10 years of age of European children born with a major CA [17].

## Methods

### Design and population

This was a European, population-based linkage cohort study. The cohort included all live births with rare structural CAs collected and validated by population-based CA registries which are members of EUROCAT (https://eu-rd-platform.jrc.ec.europa.eu/eurocat_en) [18–20].

All liveborn children with a major CA born between 1^st^ January 1995 and 31^st^ December 2014 recorded in the 13 registries of nine Western European countries were linked to mortality records up to the child’s 10^th^ birthday or to 31^st^ December 2015 (whichever was earlier), so that all live births had information on at least the first year’s survival (Table 1). Full details on the linkage methods are reported elsewhere [21–23]. A major CA is defined as an anomaly that require surgical treatment, have serious adverse effects on health or development, or have significant cosmetic impact [24].

**Table 1:** Contributing European Surveillance of Congenital Anomalies (EUROCAT) registries, included birth years, population covered, and linkage to mortality record/vital statistics

Participating registries (full registry names)	Included birth years	Birth population covered^a^	Linkage method
Denmark: Funen	1995–2014	105,770	VS
Finland	1995–2014	1,174,727	VS
France: Paris	1995–2014	597,822	VS
Italy: Emilia Romagna	2008–2014	282,094	VS
Italy: Tuscany	2005–2014	299,869	VS
Malta	1995–2014	84,737	MR
Netherlands: Northern	1995–2014	372,192	VS
Norway	1999–2014	956,939	VS
Spain: Valencian Region	2007–2014	403,099	MR
UK: East Midlands and South Yorkshire	2003–2012	717,264	VS
UK: Thames Valley	2005–2013	270,327	VS
UK: Wales	1998–2014	569,341	VS
UK: Wessex	2004–2014	325,339	VS
Total		6,159,520	

MR: registry linked to mortality record database; VS: registry linked to national/vital statistics database

^a^Extracted from the EUROCAT website: https://eu-rd-platform.jrc.ec.europa.eu/eurocat/eurocat-data/prevalence_en, accessed on 30/09/2021)

In brief, all the registries linked their CA data to vital statistics, except Malta and Valencian Region (Spain), which linked to mortality records (Table 1). Linkage to vital statistics provides information on whether the child was still alive or had died; in contrast, in a mortality database the child was assumed to be alive if no death certificate was present. Careful examination of the accuracy of the linkage was undertaken and birth years in registries during which the linkage quality was judged poor were excluded from this analysis [21]. The study period differed between registries due to different years of EUROCAT membership and because only years with high quality linkage were retained (Table 1).

### Investigated anomalies

This study focused on the survival of children with 24 different rare structural CAs, selected within EUROlinkCAT as CAs with a live birth prevalence lower than 1 per 10,000 according to the EUROCAT prevalence tables. In addition, five new congenital anomaly subgroups were defined that were not in the standard EUROCAT prevalence tables, but were assumed to have a prevalence lower than 1 per 10,000. Four of them were subsequently found to have a prevalence slightly above 1 per 10,000 when analysed in the EUROCAT database, but are included in this study. In addition, Hirschsprung’s disease, with a prevalence of 1.64 per 10,000 and pulmonary valve atresia with a prevalence of 1.01 per 10,000 are also included. Table 2 shows the 31 CAs included in this study with their livebirth prevalence estimates, together with the coding of the International Statistical Classification of Diseases and Related Health Problems version 10 (ICD-10) and version 9 (ICD-9) with the British Paediatric Association (BPA) extension used by EUROCAT to identify each anomaly [25, 26].

**Table 2:** Rare structural congenital anomalies included in the study with ICD10-BPA, ICD9-BPA and livebirth prevalence estimates with 95% confidence interval (CI)

Subgroups/anomalies	ICD10-BPA	ICD9-BPA	Prevalence per 10,000 (95% CI)
*Nervous system*			
Encephalocele	Q01	7420	0.37 (0.32–0.42)
Arhinencephaly/holoprosencephaly	Q041, Q042	74,226	0.27 (0.23–0.32)
Anomalies of corpus callosum^§^	Q040	74,221	1.74 (1.63–1.84)
*Eye*			
Anophthalmos/microphthalmos	Q110, Q111, Q112	7430, 7431	0.87 (0.80–0.95)
Anophthalmos	Q110, Q111	7430	0.17 (0.14–0.20)
Congenital glaucoma	Q150	74,320	0.39 (0.34–0.44)
*Ear, face and neck*			
Anotia	Q160	74,401	0.23 (0.20–0.28)
*Congenital heart defects*			
Common arterial truncus*	Q200	74,500	0.49 (0.44–0.55)
Double outlet right ventricle*	Q201	No code	0.78 (0.71–0.85)
Single ventricle*	Q204	7453	0.56 (0.50–0.62)
Triscuspid atresia and stenosis*	Q224	7461	0.78 (0.71–0.85)
Ebstein’s anomaly*	Q225	7462	0.50 (0.45–0.56)
Pulmonary valve atresia*	Q220	74,600	1.01 (0.93–1.09)
Hypoplastic right heart*	Q226	74,688	0.21 (0.17–0.24)
Aortic atresia/interrupted aortic arch*	Q252	74,720	0.38 (0.33–0.43)
Total anomalous pulmonary venous return*	Q262	74,742	0.68 (0.62–0.75)
*Respiratory*			
Choanal atresia	Q300	7480	0.86 (0.79–0.94)
*Digestive system*			
Hirschsprung’s disease	Q431	75,130–75,133	1.64 (1.54–1.74)
Atresia of bile ducts	Q442	75,165	0.40 (0.35–0.45)
Annular pancreas	Q451	75,172	0.25 (0.21–0.29)
Anomalies of intestinal fixation^§^	Q433	7514	1.12 (1.04–1.21)
*Urinary*			
Unilateral renal agenesis^§^	Q600	No code	2.00 (1.90–2.12)
Accessory kidney^§^	Q630	75,330	1.49 (1.39–1.58)
Bladder exstrophy^¥^	Q641	7535	0.31 (0.27–0.36)
Epispadia^¥^	Q640	75,261	0.30 (0.26–0.35)
Posterior urethral valves^¥^	Q6420	75,360	0.92 (0.84–1.00)
Prune belly sequence^¥^	Q794	75,672	0.08 (0.06–0.10)
*Genital*			
Indeterminate sex	Q56	7527	0.51 (0.45–0.56)
*Other anomalies*			
Situs inversus	Q893	7593	0.55 (0.49–0.61)
VATER/VACTERL	Q8726	759,895	0.26 (0.22–0.30)
Arthrogryposis multiplex congenita^§^	Q743	75,580	0.50 (0.45–0.56)

*All listed congenital heart defects are part of the severe congenital heart defects subgroup as defined in EUROCAT Guide 1.4 [18]

^§^ New subgroups defined within EUROlinkCAT

^¥^Original EUROCAT-subgroups were: Bladder exstrophy and/or epispadias and Posterior urethral valves and/or prune belly

ICD10-BPA, International Classification of Diseases version 10 with the British Paediatric Association extension; ICD9-BPA, International Classification of Diseases version 9 with the British Paediatric Association extension

### Statistical analysis

The analyses were based on standardized EUROCAT variables together with a common data model developed for EUROlinkCAT to standardize the local variables obtained from the linkage [17]. Such standardization allowed centrally written syntax scripts to be developed both for checking the quality of data linkage and for analysing the data to be run by all participating registries [17, 21].

To account for censoring of individuals due to emigration or reaching the study end date before reaching the 10th birthday, Kaplan–Meier survival analyses were performed within each registry by running centrally written syntax scripts. The survival estimates with 95% confidence intervals (CI) together with the number at risk (alive at the beginning of each time period) and the number of deaths in each time period for each CA subgroup were uploaded by each registry to the CRR at Ulster University (UK) and then transferred to the research team using a secure web platform. No individual case data were shared.

The Kaplan–Meier survival estimates from each registry were then combined centrally in a random-effects meta-analysis of the survival at five ages separately (1 week, 4 weeks and 1, 5 and 10 years) to estimate the overall survival for each CA.

Similarly, 10-year survival estimates conditional on having survived at 4 weeks calculated for each registry were combined in a random-effects meta-analysis.

Differences between registries were evaluated for eight rare CAs, where each had at least 500 live births at risk, by plotting the forest plot of the meta-analysis of the survival at 5 years and reporting the I^2^ statistic as a measure of the observed between-registry heterogeneity.

All statistical analyses were performed using Stata16 (StataCorp LP, College Station, TX, USA).

## Results

Thirteen European registries from nine countries, covering a population of 6,159,520 births in 1995–2014 were included in the study, with 5 out of 13 registries covering all 20 birth years (Table 1). There was a total of 12,685 liveborn children with one of the 31 rare structural CAs (Table 3).

**Table 3:** Survival estimates (with 95% confidence intervals, CI) for selected age groups up to 10 years of age and survival at 10 years conditional on surviving at 4 weeks, for children born with a rare structural congenital anomaly in 13 EUROCAT registries in nine Western European countries

Congenital anomaly groups and subgroups	No. of live births	No. of deaths up to 10 years	1 week (95% CI)	4 weeks (95% CI)	1 year (95% CI)	5 years (95% CI)	10 years (95% CI)	10-year conditional on surviving to 4 weeks (95% CI)
*Nervous system*								
Encephalocele	228	55	88.8 (81.9–96.3)	87.9 (80.9–95.4)	83.5 (76.9–90.7)	81.6 (74–7-89.1)	79.2 (72.1–87.0)	94.8 (88.3–97.7)
Arhinencephaly/holoprosencephaly	167	105	58.1 (44.3–76.2)	55.6 (42.5–72.9)	47.4 (36.4–61.6)	40.4 (27.4–59.5)	35.6 (22.2–56.9)	77.8 (58.9–88.7)
Anomalies of corpus callosum	1069	250	94.2 (91.9–96.6)	89.2 (86.3–92.3)	83.2 (79.8–86.9)	78.2 (75.1–81.5)	77.0 (73.5–80.6)	92.4 (90.8–93.8)
*Eye*								
Anophthalmos/microphthalmos	536	139	89.2 (83.6–95.3)	86.2 (80.1–92.9)	80.9 (74.5–87.9)	79.4 (72.5–86.9)	77.4 (70.6–84.9)	94.5 (91.6–96.5)
Anophthalmos	103	28	85.1 (69.7–100.0)	83.8 (69.3–100.0)	80.8 (65.8–99.1)	76.4 (58.3–99.9)	73.4 (58.3–92.4)	93.4 (74.8–98.4)
Congenital glaucoma	238	12	99.8 (99.3–100.0)	99.4 (98.4–100.0)	97.6 (95.2–100.0)	96.3 (92.6–100.0)	94.6 (89.5–100.0)	96.2 (89.1–98.7)
*Ear, face and neck*								
Anotia	144	11	94.9 (89.2–100.0)	94.7 (88.9–100.0)	92.2 (86.7–97.9)	91.9 (86.4–97.7)	91.7 (86.0–97.7)	99.8 (98.2–100.0)
*Congenital heart defects*								
Common arterial truncus	301	115	91.1 (87.3–94.9)	78.7 (73.8–83.9)	63.9 (57.3–71.3)	61.4 (55.0–68.5)	60.5 (53.7–68.2)	95.0 (89.0–97.8)
Double outlet right ventricle	481	115	95.8 (91.7–100.0)	91.9 (87.2–96.8)	82.9 (78.8–87.3)	79.5 (74.7–84.6)	78.0 (73.3–83.0)	92.5 (88.4–95.2)
Single ventricle	344	106	90.5 (85.4–96.0)	86.4 (80.4–92.8)	75.8 (67.2–85.5)	72.1 (63.2–82.3)	70.9 (61.7–81.4)	93.4 (89.0–96.7)
Tricuspid atresia and stenosis	479	120	92.7 (89.6–95.9)	86.4 (82.8–90.3)	80.4 (76.3–84.7)	77.9 (73.2–82.9)	77.0 (72.6–81.6)	94.6 (92.4–96.3)
Ebstein’s anomaly	309	57	92.2 (88.4–96.3)	86.5 (81.1–92.2)	81.0 (73.8–89.0)	78.9 (70.9–87.8)	78.1 (69.7–87.5)	95.4 (87.8–98.3)
Pulmonary valve atresia	622	189	95.9 (93.0–98.8)	89.2 (86.0–92.5)	80.0 (75.4–84.9)	76.2 (70.9–81.8)	73.6 (68.4–79.1)	90.6 (87.5–92.9)
Hypoplastic right heart	127	35	91.9 (86.3–97.8)	82.8 (75.3–90.9)	72.9 (64.7–82.2)	72.7 (64.4–82.0)	72.1 (63.8–81.5)	99.1 (90.5–99.9)
Aortic atresia/interrupted aortic arch	234	75	90.8 (86.8–95.0)	77.7 (68.3–88.4)	64.5 (52.8–78.8)	63.0 (51.3–77.5)	62.8 (51.1–77.1)	95.7 (87.6–98.5)
Total anomalous pulmonary venous return	419	117	94.0 (90.8–97.2)	87.3 (82.8–91.9)	78.2 (72.4–84.3)	76.4 (70.7–82.4)	75.1 (69.4–81.2)	94.4 (91.4–96.3)
*Respiratory system*								
Choanal atresia	532	81	96.4 (93.6–99.3)	94.5 (90.8–98.4)	90.1 (87.4–94.8)	88.7 (85.1–92.5)	88.4 (84.8–92.1)	96.8 (94.4–98.1)
*Digestive system*								
Hirschsprung’s disease	1008	45	99.8 (99.6–100.0)	99.5 (99.0–100.0)	98.2 (97.3–99.1)	97.1 (95.6–98.4)	96.6 (95.3–97.9)	97.2 (96.2–97.9)
Atresia of bile ducts	246	46	99.3 (98.2–100.0)	98.5 (96.8–100.0)	89.3 (94.3–84.6)	84.5 (78.7–90.8)	84.1 (77.9–90.8)	90.8 (81.8–95.4)
Annular pancreas	153	9	97.5 (97.5–100.0)	95.3 (91.2–99.6)	92.7 (88.4–97.2)	91.6 (87.0–96.4)	89.9 (83.5–96.9)	98.8 (88.1–99.9)
Anomalies of intestinal fixation	689	107	95.8 (92.4–99.2)	93.4 (89.4–97.6)	90.4 (85.9–95.1)	89.5 (85.1–94.2)	89.2 (84.9–93.6)	98.5 (97.5–99.1)
*Urinary system*								
Unilateral renal agenesis	1237	92	96.3 (94.8–97.8)	95.7 (93.9–97.6)	94.4 (92.2–96.7)	93.9 (91.5–96.4)	93.4 (90.6–96.4)	97.6 (95.5–98.8)
Accessory kidney	915	16	99.9 (99.8–100.0)	99.6 (99.1–100.0)	99.1 (98.3–99.8)	99.0 (98.2–99.8)	99.0 (98.2–99.8)	99.3 (98.4–99.7)
Bladder exstrophy	190	17	97.6 (94.6–100.0)	96.0 (91.5–100.0)	93.2 (88.4–93.2)	92.6 (87.6–97.9)	92.6 (87.6–97.9)	98.8 (95.6–99.7)
Epispadias	185	2	99.5 (98.5–100.0)	99.5 (98.5–100.0)	99.5 (98.5–100.0)	99.5 (98.5–100.0)	99.5 (98.5–100.0)	99.8 (98.8–99.9)
Posterior urethral valves	566	51	95.0 (91.9–98.2)	94.4 (91.2–97.8)	93.4 (89.9–97.0)	93.3 (89.9–96.9)	93.3 (89.9–96.9)	99.2 (97.9–99.7)
Prune belly sequence	48	17	76.4 (52.9–100.0)	75.3 (52.4–100.0)	67.0 (43.2–100.0)	59.0 (35.8–97.1)	57.4 (34.3–96.1)	95.2 (78.9–99.0)
*Genital*								
Indeterminate sex	311	73	88.8 (82.5–95.6)	84.6 (77.7–92.1)	80.4 (71.9–89.9)	79.3 (70.5–89.2)	78.6 (69.5–88.8)	95.6 (92.6–97.4)
*Other anomalies*								
Situs inversus	337	44	97.1 (95.1–99.1)	95.5 (93.0–98.1)	91.6 (87.9–95.4)	90.3 (86.4–94.3)	89.6 (85.9–93.6)	97.3 (95.3–98.4)
VATER/VACTERL	157	33	93.6 (87.2–100.0)	92.2 (86.1–98.7)	85.9 (80.0–92.3)	82.6 (76.7–89.1)	80.9 (74.3–88.1)	94.4 (86.1–97.8)
Arthrogryposis multiplex congenita	310	104	85.6 (79.6–92.0)	81.2 (75.0–88.0)	73.6 (66.6–81.4)	70.4 (63.1–78.6)	69.4 (62.0–77.6)	89.8 (84.9–93.2)

10-years survival conditional on surviving at 4 weeks was calculated for 12 registries. CHD, congenital heart defect; CI, confidence interval

Prune belly sequence and anophthalmos were the rarest investigated CAs with only 48 and 103 live births respectively and unilateral renal agenesis the most common with 1237 live births (Table 3).

Table 3 shows the survival estimates (with 95% CIs) calculated for each rare CA. As expected, there was considerable variation in survival between individual anomalies.

At 1 week, only children with arhinencephaly/holoprosencephaly and prune belly sequence had survival below 80%, being 58.1% (95% CI: 44.3–76.2) and 76.4% (95% CI: 52.9–100.0) respectively.

Ten-year survival varied from 35.6% (95% CI: 22.2–56.9) for children with arhinencephaly/holoprosencephaly to 99.5% (95% CI: 98.5–100.0) for children with epispadias.

Ten-year survival was below 80% for 17 CAs, which included all nine severe rare congenital heart defects (CHDs). In particular, children with common arterial truncus and aortic atresia/interrupted aortic arch had 10-year survival lower than 65%.

Children with prune belly sequence had consistently low survival across all age points, declining from 76.4% (95% CI: 52.9–100.0) at 1 week to 67.0% (95% CI: 43.2–100.0) at 1 year and further to 57.4% (95% CI: 34.4–96.1) at 10 years of age.

In general, children with rare CAs of the digestive and urinary system, with the exception of prune belly sequence, had a relatively high survival at all five age points.

**Fig. 1:**
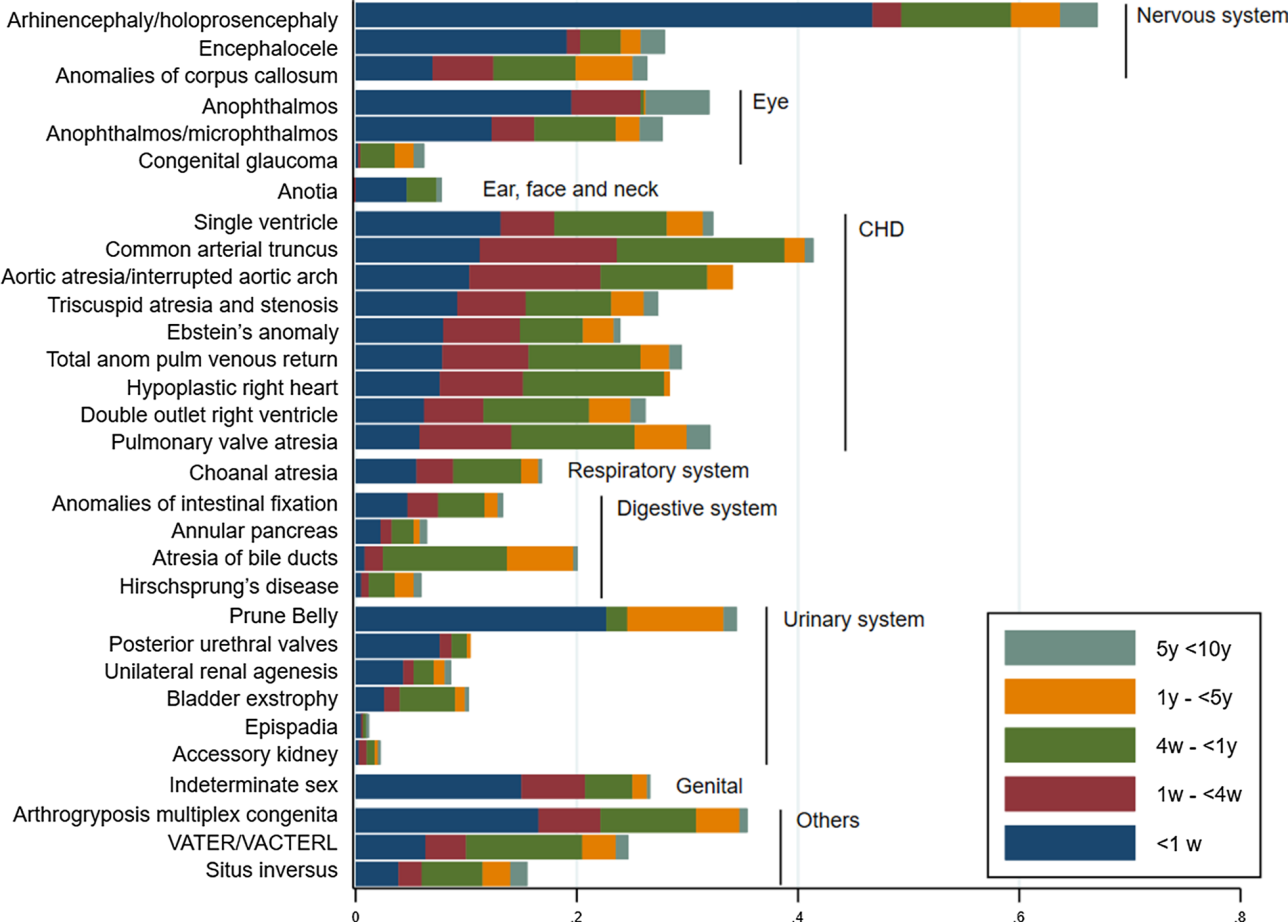
Proportion of deaths at 1 week (1 w) of age, between 1 and 4 weeks (4 w), between 4 weeks and 1 year (1 y), between 1 and 5 years (5 y), and between 5 and 10 years (10 y) for the rare structural congenital anomalies sorted according to decreasing proportion of deaths at 1 week of age and showed by group

Figure 1 shows the proportion of deaths for each CA at each age by group of CA. For children with arhinencephaly/holoprosencephaly, encephalocele, prune belly sequence, posterior urethral valve, anotia, anophthalmos, and indeterminate sex more than 50% of deaths occurred within the first week. In general, for children with rare CHDs more than 50% of deaths occurred within the first month. Children with anomalies of the digestive or urinary system had a much higher proportion of deaths occurring at later ages.

The 10-year conditional survival estimates (i.e. the survival at 10 years of age of children who have survived at 4 weeks), are all above 90% (Table 3), with the exception of children with arthrogryposis multiplex congenita (89.8%) and arhinencephaly/holoprosencephaly (77.8%). For 17 out of 31 CAs, the 10-year conditional survival was higher than 95%.

**Fig. 2:**
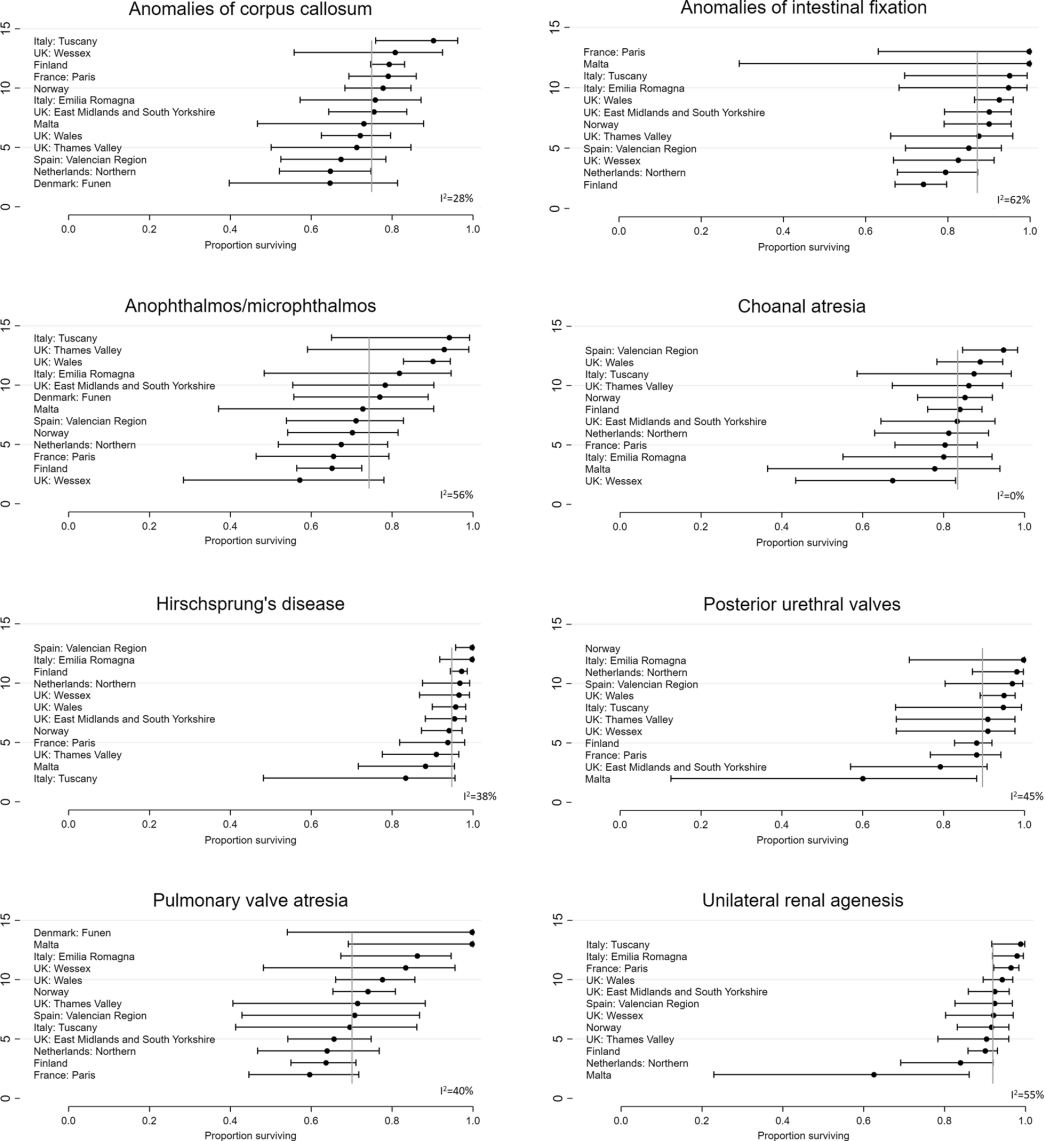
Five-year survival estimates (with 95% Confidence Intervals (CI)) by registry (birth year period: 1995–2014) and the I^2^ statistic as a measure of the observed between-registry heterogeneity calculated by a random effect meta-analysis on eight rare CAs with at least 500 liveborn cases. d = days; w = week, m = months, y = years

Figure 2 shows the differences in the survival at 5 years among registries for eight rare CAs with at least 500 live births. The greatest heterogeneity among registries (I^2^ > 50%) was observed for the subgroups anophthalmos/microphthalmos, anomalies of intestinal fixation and unilateral renal agenesis. A moderate heterogeneity between registries was observed for all the other rare structural CAs, with the exception of choanal atresia for which the survival appeared almost homogeneous across the investigated areas.

## Discussion

This study reports the survival of children born with rare structural CAs in western Europe, using population-based data on children born in the period 1995–2014 linked to mortality data.

There are few studies on population-based long-term survival of children with rare structural CAs, therefore it is not possible to make a direct comparison between our results and other published studies. Most of the studies on rare CAs are hospital-based series mainly reporting fatality rates rather than survival estimates in live births.

The rare CAs investigated in this study are heterogeneous and can be associated with different complex conditions, which accounts for the considerable variation in survival observed between individual anomalies. In addition, the proportion of associated anomalies might differ substantially by type of CA: it has been reported that 32–34% of the respiratory and of eye, face and neck CAs most likely occur with other CAs and that CHDs, limb, and genital are the least likely to occur with other CAs (12–13%) [27]. This different proportions may have an impact on survival mainly for the less severe anomalies [28, 29].

Children with arhinencephaly/holoprosencephaly had the lowest survival at all five investigated ages with only 58.1% of children surviving the first week of life. Holoprosencephaly is a brain malformation characterized by four forms (in decreasing order of severity: alobar, semilobar, lobar and middle interhemispheric variant) with the alobar form present approximately in two out of three individuals, thus probably explaining the observed low survival [30].

Children with prune belly sequence also had a low survival in the first week of life (76.4%). Prune belly sequence can be characterized by a wide variability in severity and clinical manifestations, such as abdominal muscle deficiency, lung and renal dysfunction, associated congenital heart defects, and cryptorchidism [31].

Children with arthrogryposis multiplex congenita (AMC), a CA that can be associated with multiple developmental defects and be part of a large number of syndromes with or without central nervous system involvement, also showed one of the lowest survival estimates both in the first week (85.6%) and at 10 years (69.4%). A EUROCAT population-based study on AMC including the birth period 1980–2006 reported that 23% of children had died within the first week of life. The higher mortality observed in that study may have been due to the earlier time period studied or bias arising from missing data on first week survival [29].

In our study, all children with rare CHDs had 10-year survival lower than 80%. All the rare CHDs here investigated are severe, commonly requiring cardiac surgery in the first year of life. Some of them, such as common arterial truncus, total anomalous pulmonary venous return and single ventricle, are incompatible with survival without a surgery early in infancy. A meta-analysis by Best and Rankin [32] investigating the long-term survival of children born with CHDs reported pooled 5-year survival estimates of 47.4%, 59.8%, 61.2% and 65.6% for common arterial truncus, single ventricle, total anomalous pulmonary venous return and Ebstein’s anomaly respectively, compared to survival of 61.4%, 72.1%, 76.4% and 78.9% estimated in our study. The lower meta-analytic estimates observed by Best and Rankin may be in part attributed to their cohorts encompassing earlier periods (birth years starting in the 1970s–1980s) and the inclusion of diverse study designs, CHD classification, geographical areas and mortality sources.

Children with atresia of bile ducts almost always survive the first week of life (99.3%). The diagnosis is usually given 2–6 weeks after birth [33]. However, survival estimates at 5 and 10 years of age (84.5% and 84.1%, respectively) in children with this severe CA, which is incompatible with life if not operated early in infancy, are still relatively low despite a wider use of liver transplantation for these patients in recent decades. Our survival estimates are comparable with pooled estimates in a systematic review of children with biliary atresia (85% and 82% for 5- and 10-year survival respectively) [6].

For children with Hirschsprung’s disease and anomalies of intestinal fixation, mortality may be due to enterocolitis and acute complications with intestinal ischemia [34].

Mortality of children with anophthalmos and microphthalmos is most likely explained by associated anomalies such as severe cerebral anomalies and/or lethal chromosomal anomalies [35]. Similarly, survival estimates lower than 100% for children with congenital glaucoma can be explained by associated anomalies or genetic diseases [36].

In children with situs inversus, survival of 91.6% was observed at 1 year (89.6% at 10 years) which is probably due to the presence of associated severe CHDs such as transposition of great vessels [37].

In general, for rare CAs, the number of live births is too small to evaluate geographical differences across different registries, but eight investigated anomalies with more than 500 children were considered suitable for a meta-analysis aimed at evaluating regional variations. However, we observed, as expected, a lower precision of the estimates for some registries in some of these rare CAs due to small numbers of events. The results showed a moderate variation in survival between participating registries that is in full agreement with what was reported in a methodological study on the geographical variation in survival showing a high variability only for major subgroups of CAs [22]. The low/moderate heterogeneity observed in our study suggests consistency and generalizability of our results and, as a consequence, accurate survival estimates for the rare CAs investigated.

### Study strengths and limitations

The main strength of the study is that the pooling of data from high-quality population-based specialized EUROCAT registries from across Europe resulted in the largest cohort of children born with rare structural CAs at European level to date. This allowed reliable survival estimates up to 10 years of age to be produced. Another strength was the use of standardized approaches in EUROCAT registries (data collection, coding and classification) and in EUROlinkCAT (standardising variables to a common data model) which enabled common syntax scripts to produce standardised analytic results.

A limitation of the study is that isolated cases of each rare CA could not be analysed due to the extremely small sample sizes in the registries. However, as the data is from a population-based cohort, the presence of associated anomalies reflects the expected occurrence of anomalies in future births and therefore the survival rates can be considered an unbiased estimate of predicted survival for children with these rare anomalies. A second limitation is that all registries report cases diagnosed within 1 year of age, but some of the investigated rare CAs (e.g. anomalies of intestinal fixation, unilateral renal agenesis, accessory kidney, situs inversus if not picked up by ultrasound scans) may be diagnosed later and children with these anomalies may not be included in the study; this is particularly true for less severe cases without associated anomalies. For this reason, there might be an overestimate of mortality for some rare CA due to the exclusion of less severe cases diagnosed after infancy. Another limitation is the lack of registries from Central and Eastern Europe. Three EUROCAT member registries from Central and European Europe participated in EUROlinkCAT, but we excluded them from the analysis: two of them were excluded due to low quality data linkage and one due to extremely low survival rates. As other studies have also found higher infant mortality in Eastern Europe [38] it was decided that including only one Eastern European country would not enable us anyway to produce survival estimates referred to the whole Europe. For these reasons, the results of our study should be inteded as representative of Western Europe only.

Finally, due to the small sample sizes occurring in each registry it was not possible to investigate any association between survival and socio-economic status that might provide useful information for making inferences about the quality of care provided to children born with CAs.

## Conclusions

This multi-centre population-based European study provided a sufficiently large, standardized cohort to produce reliable survival estimates of children with rare structural anomalies up to 10 years of age born in the period 1995–2014. There was considerable variation in survival for children with the different anomalies, with only moderate variability between registries. For the majority of anomalies, more than 50% of deaths occurred within the first month and the 10-year survival conditional on surviving the first four weeks of life was above 95%. Having reliable information on long-term survival of children born with specific CAs is of major importance for the health professionals involved in counseling parents, especially when facing a prenatal diagnosis of a rare CA.

## Data Availability

The data that support the findings of this study are available from the participating registries of congenital anomalies, but restrictions apply to the availability of these data, which were used under license for the current study, and so are not publicly available. Data are however available from the authors for scientifically valid requests and with permission of the participating registries of congenital anomalies.

## Abbreviations

AMC: Arthrogryposis multiplex congenita
CAs: Congenital anomalies
CHDs: Congenital heart defects
CI: Confidence interval
CRR: Central Results Repository
ICD-10: International Classification of Diseases version 10
